# Choroidal Vascularity Index in Schizophrenia Compared to Healthy Subjects: A Cross‐Sectional Study

**DOI:** 10.1002/hsr2.72048

**Published:** 2026-04-11

**Authors:** Mehrdad Motamed Shariati, Maryam Naghib, Marziyeh Fotouhi, Mohammad Reza Fayyazi Bordbar

**Affiliations:** ^1^ Eye Research Center Mashhad University of Medical Sciences Mashhad Iran; ^2^ Psychiatry and Behavioral Sciences Research Center Mashhad University of Medical Sciences Mashhad Iran

**Keywords:** biomarker, choroidal vascularity index, inflammation, ophthalmic imaging, schizophrenia

## Abstract

**Background and Aims:**

This study aims to evaluate choroidal vascular architecture in patients with schizophrenia compared to healthy individuals.

**Methods:**

This cross‐sectional study was conducted between 2019 and 2021. In total, 22 hospitalized patients with documented instances of schizophrenia and 22 healthy subjects were included. Demographic, clinical ophthalmic, psychiatric, and enhanced‐depth imaging optical coherence tomography (EDI‐OCT) data were gathered. The choroidal luminal area (LA), total choroidal area (TCA), and choroidal thickness (CT) were calculated using the ImageJ software. The choroidal vascularity index (CVI) was calculated as the ratio of LA to TCA.

**Results:**

The mean ± standard deviation (SD) of the LA was 0.301 ± 0.03 mm^2^ and 0.251 ± 0.04 mm^2^ in the schizophrenia and healthy subjects, respectively (*p* < 0.001). Besides, TCA was significantly larger in schizophrenia patients (*p* = 0.01). The results showed that despite no statistically significant difference in the subfoveal choroidal thickness (SFCT) between the two groups of schizophrenia and healthy subjects, CVI is significantly higher in schizophrenia patients (*p* = 0.04).

**Conclusion:**

Increased CVI supports the hypothesis of microvascular dysfunction in schizophrenia. However, the precise mechanism and pathogenesis of microvascular changes in schizophrenia are unclear.

## 1 Introduction

Schizophrenia is a chronic progressive mental illness with a spectrum of positive, negative, and cognitive symptoms [1]. Although the exact pathophysiology is unclear, genetic, neuroinflammatory, neurodevelopmental, and neurodegenerative mechanisms have been proposed to be associated with the pathophysiological process [2, 3]. Several morphological alterations, such as abnormalities of the white matter fibers, expansion of the ventricles, and disruption of the frontotemporal, thalamocortical, and subcortical‐limbic circuits, have been reported in several structural investigations of schizophrenia [4, 5, 6].

The genetic‐vascular‐inflammatory theory of schizophrenia, according to Hanson and Gottesman, can account for the variety of symptoms [7]. According to this theory, environmental triggers such as infection, trauma, or hypoxia can cause inflammatory reactions in a genetically sensitive patient, harming the brain's microvessel–astrocyte coupling system and resulting in psychoses [8, 9]. Neuroimaging studies have shown unusual cerebral blood flow, a measure of cerebrovascular functioning, in psychotic patients. Also, vascular remodeling, hypoxia signaling, and aberrantly forming vascular networks are risk factors for schizophrenia [10, 11, 12].

In recent years, a great deal of attention has been paid to the functional and structural neuroimaging of schizophrenia to better understand the underlying neurobiology and generate accurate diagnostic biomarkers. Considering the embryonic origin and common blood supply of the retina and the brain, in recent years, researchers have used the retina as an evaluable model to investigate neurodegenerative processes. The study of vascular and nerve structures of the retina and optic nerve with the optical coherence tomography angiography method has provided valuable data in this field [13]. Most of the previous studies have shown a decrease in macular vascular density in schizophrenia compared to the healthy population [14, 15]. However, very few studies have been conducted in the field of choroidal blood flow changes in these patients, and the limitation of these studies is the assessment of choroidal thickness (CT) without considering the luminal and stromal components of its vessels. A few investigations revealed no distinctions between healthy participants and patients with schizophrenia in terms of choroid vascular density or CT results [16]. Enhanced‐depth imaging optical coherence tomography (EDI‐OCT) allows for assessing the choroid's thickness and vasculature. The choroidal vascularity index (CVI), a novel quantitative metric, was proposed by Agrawal and colleagues to evaluate the vasculature of the choroid in both healthy and diseased eyes. CVI is defined as the proportion of the choroidal luminal area (LA) to the total choroidal area (TCA) [17, 18]. Various techniques have been put forth to calculate the CVI. One of the most popular image‐processing software to calculate CVI is ImageJ (freely given by the National Organization of Wellbeing, Bethesda, MD, USA; http://imagej.nih.gov/ij/) [19].

Recent studies have increasingly highlighted the significance of the choroid as a key neurovascular interface that may mirror or interact with central nervous system (CNS) barriers such as the blood–brain barrier (BBB) and the blood–cerebrospinal fluid barrier (BCSFB). The choroid's dense vasculature and immunological properties position it as a potential peripheral window into CNS processes. Schizophrenia, a complex neuropsychiatric disorder, has been linked to subtle neurovascular dysregulations, including those involving barrier integrity and neuroimmune interactions. Intriguingly, recent findings suggest that alterations in the choroidal plexus may reflect systemic manifestations of CNS dysfunction in schizophrenia [20, 21]. Moreover, both the BBB and the BCSFB exhibit structural and functional similarities with the outer and inner blood–retinal barriers, which are anatomically proximate to the choroid. These barriers share molecular transport mechanisms and immunoregulatory functions often disrupted in schizophrenia. Such disruptions may permit aberrant peripheral–central–peripheral signaling loops, facilitating neuroinflammation, oxidative stress, or immune cell infiltration across traditionally immune‐privileged compartments [22, 23, 24]. Consequently, the choroid may not only serve as a biomarker of CNS integrity but also play a more active role in the pathophysiology of schizophrenia by bridging peripheral and central compartments. This emerging understanding underscores the relevance of studying the choroid in the context of schizophrenia and neurovascular disorders more broadly.

Although the literature remains limited, recent studies have begun to explore choroidal structural changes in patients with schizophrenia. Demirlec and colleagues investigated choroidal structure and vascularity using EDI‐OCT in individuals with first‐episode psychosis, those at ultra‐high risk for psychosis, and healthy controls. While no significant differences were found in CT, the luminal choroidal area to stromal choroidal area (LCA/SCA) ratio and the CVI significantly differed across groups [25].

Given the choroid's structural and functional overlap with CNS barriers and its emerging role in neuroinflammatory and neurovascular regulation, we aimed to investigate whether schizophrenia is associated with measurable alterations in choroidal vasculature. This study seeks to contribute to the growing literature on peripheral biomarkers of CNS dysfunction in psychiatric disorders.

## 2 Methods

### 2.1 Participants

This cross‐sectional study was carried out in the Ibn Sina Psychiatric Hospital in Mashhad, Iran, between 2019 and 2021. We included 22 hospitalized patients with documented instances of schizophrenia who were receiving risperidone as an antipsychotic. To confirm the diagnosis, two psychiatrists assessed all the cases applying the DSM‐5 criteria. Inclusion criteria were as follows: age between 18 years or more, clinical diagnosis of schizophrenia according to DSM‐5, and stable antipsychotic regimen for at least [e.g., 4 weeks] before enrollment. Exclusion criteria were as follows: history of ocular trauma or surgery, the use of specific drugs (4‐aminoquinolines, amiodarone, calcium channel blockers, alcohol) that affect choroidal blood flow, presence of any other major psychiatric disorder (e.g., bipolar disorder, major depressive disorder), neurological disease (e.g., epilepsy, multiple sclerosis), diabetes mellitus, uncontrolled hypertension, glaucoma, or any systemic or ocular disease known to affect the choroid, current substance abuse or dependence, refractive error greater than ±6.00 diopters, and poor quality ocular imaging (e.g., due to media opacities or noncooperation). We categorized the individuals with schizophrenia into two subgroups: DITYL (illness duration < 2 years) and DIMTY (illness duration > 2 years). Alongside the patients' demographic data (age and gender), information on their medical history, prescriptions, and mental health status was noted.

The Positive and Negative Syndrome Scale (PANSS) was employed in this investigation to assess the severity of the disease. To evaluate the severity of symptoms among individuals with schizophrenia, PANSS was created in 1986. This test uses 30 items to assess 3 different areas of symptoms associated with schizophrenia (positive and negative symptoms, and general psychopathology, including 7, 7, and 16 items, respectively). Every item has a maximum score of 7. Based on the PANSS total score, we classified the patients with schizophrenia into four groups: mildly ill (up to a PANSS score of 58), moderately ill (59–75), markedly ill (76–95), and severely ill (96–116).

### 2.2 Ethical Considerations

The Ethics Committee of Mashhad University of Medical Sciences granted clearance for this study, which followed the Declaration of Helsinki (Approval Number: IR.MUMS.MEDICAL.REC.1399.696). Either the patient or their legal guardians gave us their informed consent.

### 2.3 Ophthalmic Examination and Imaging

At the Khatam Al‐Anbia Eye Hospital, all subjects underwent the following ophthalmic evaluations: macular EDI‐OCT (AngioVue RTVue XR Avanti, Optovue, Fremont, CA, USA, software version 2018.0.0.18) with 3 × 3 mm scan size, slit‐lamp biomicroscopy, Goldmann applanation tonometry, and best‐corrected visual acuity measurement with thumbing E chart. After discarding any images with a quality index of less than 6/10, the imaging process was repeated. The resulting data from that eye were removed from the analysis in the event of any major anomalies in ophthalmic assessments (visually significant cataract, epiretinal membrane, etc.) or two imaging scans with quality indices lower than 6/10. The control group consisted of 22 healthy individuals with no history of mental, systemic, or ocular diseases. None of the participants had used medications that would have affected the choroidal circulation. The groups were comparable in terms of age and sex distribution, with no statistically significant differences between them. Both eyes were imaged using the same protocol. Initial comparisons showed no significant differences in choroidal measurements between eyes. One eye per participant was randomly selected for the final analysis to maintain statistical independence and avoid bias due to within‐subject correlation.

### 2.4 Image Processing

**Figure 1:**
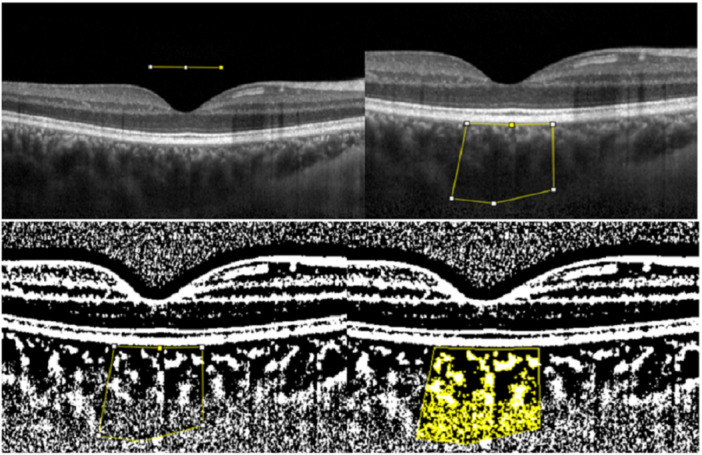
CVI calculation using ImageJ software. Left upper: the yellow line represents the 1500 microns centered on the fovea. Right upper: the yellow rectangle represents the region of interest (ROI) limited with RPE superiorly and chorioscleral junction inferiorly. The area of ROI is defined as the TCA. Left lower: image binarization using the Niblack algorithm. Right lower: calculating the LA in the ROI.

To assess the CT, EDI‐OCT was used. The region between the choroidal–scleral junction and the external border of the retinal pigmented epithelium (RPE) was defined as subfoveal choroidal thickness (SFCT). The National Organization of Wellbeing, Bethesda, MD, USA, freely provided the ImageJ software (http://imagej.nih.gov/ij/), which was used to quantify the CVI. With the software's default alternative, an 8‐bit image was created from an EDI‐OCT B‐scan image. The TCA at 750 microns on each side of the foveal center was determined by manually creating the lower border at the choroid–sclera intersection and the upper border at the RPE–Bruch's complex. To restrict the choroidal LA and stromal area (SA), we used the Niblack auto local threshold algorithm as the image adjustment technique (Figure 1). CVI was calculated as the ratio of LA to TCA [26, 27]. Manual segmentation of the choroidal boundaries was performed using ImageJ by an experienced grader who was blinded to the clinical status (schizophrenia or healthy control) of the participants.

### 2.5 Statistical Analysis

For statistical analysis, we utilized the Statistical Package for Social Sciences (SPSS) software version 22 (IBM SPSS Statistics, IBM Corporation, Chicago, IL). The Shapiro–Wilk test was employed to verify the normal distribution of the data. To confirm that the two groups were matched, the independent samples *t*‐test and *χ* ^2^ test were employed. The statistical significance of the difference in the TCA, LA, SA, CVI, and CT between patients with schizophrenia and controls was determined by independent samples *t*‐test or its nonparametric equivalent. We used Pearson's correlation to investigate the relationship between PANSS and choroidal parameters, and ANOVA plus post hoc analysis to compare choroidal vascular parameters in subgroups of schizophrenia patients based on PANSS scores and the control group. We considered a *p* < 0.05 as the level of statistical significance.

## 3 Results

A total of 22 patients with schizophrenia (11 males and 11 females) and 22 healthy individuals (12 males and 10 females) participated in the study. The mean ± standard deviation (SD) age of the healthy and individuals with schizophrenia was 34.36 ± 6.56 years and 35.86 ± 9.29 years, respectively (*p* = 0.540) (Table 1). Data on PANSS scores were collected from 22 individuals (schizophrenia group). The PANSS score had a mean ± SD of 83.29 ± 13.77 and ranged from 53 to 116, exhibiting a normal distribution (Table 2). There were 2, 6, 12, and 2 cases in each of the 4 categories of schizophrenia: mildly ill, moderately ill, markedly ill, and severely ill. Eight cases of DITYL and 14 cases of DIMTY were found among the patients with schizophrenia. A mean ± SD dosage of 4.40 ± 1.40 mg of risperidone (approximately equivalent to 440 ± 140 mg/day of chlorpromazine) was administered to every patient with schizophrenia. To alleviate agitation, some patients occasionally used anticholinergics, benzodiazepines, and other antipsychotics. Three schizophrenia patients reported smoking cigarettes, while none of the participants in the control group did (*p* = 0.73).

**Table 1:** Demographic information.

		Schizophrenia	Control	*p*
Sex	Male	11	12	0.763
	Female	11	10	
Age	Mean (SD)	35.86 ± 9.29	34.36 ± 6.56	0.540

**Table 2:** PANSS score of patients with schizophrenia.

PANSS scale	Mean	SD
Positive	19.48	4.78
Negative	20.48	5.36
General psychopathology	43.48	6.67
Anergia	10.43	3.30
Thought disorder	10.90	2.74
Activation	5.38	1.96
Paranoid/belligerence	9.95	2.11
Depression	10.05	2.16
PANSS total score	83.29	13.77

**Figure 2:**
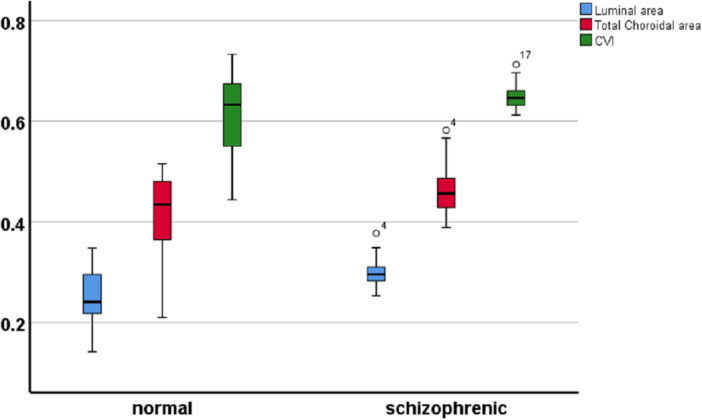
Choroidal vascular parameters in two groups of schizophrenia and healthy subjects.

Comparing the choroidal vascular parameters between the two groups showed a statistically significant difference in the LA, TCA, and CVI. The mean ± SD of the LA was 0.301 ± 0.03 mm^2^ and 0.251 ± 0.04 mm^2^ in the schizophrenia and healthy subjects, respectively (*p* < 0.001). Besides, TCA was significantly larger in schizophrenia patients (*p* = 0.01). The results showed that despite no statistically significant difference in the SFCT between the two groups of schizophrenia and healthy subjects, CVI is significantly higher in schizophrenia patients (*p* = 0.04) (Table 3 and Figure 2).

**Table 3:** Comparisons of choroidal vascular parameters between patients with schizophrenia and controls.

	Group	Mean ± SD	*p*
Luminal area (LA) (mm^2^)	Schizophrenia	0.301 ± 0.03	0.000†
	Control	0.251 ± 0.04	
Total choroidal area (TCA) (mm^2^)	Schizophrenia	0.466 ± 0.05	0.01†
	Control	0.417 ± 0.07	
Choroidal vascularity index (CVI)	Schizophrenia	0.648 ± 0.02	0.04†
	Control	0.609 ± 0.09	
Subfoveal choroidal thickness (SFCT) (µm)	Schizophrenia	312.93 ± 18.13	0.443
	Control	305.18 ± 43.13	

^†^ Considered statistically significant.

Regarding the severity of schizophrenia, we found that LA is significantly higher in markedly ill and moderately ill patients than in healthy participants. Due to the small number of cases in each subgroup of mildly ill and severely ill patients, we did not include them in the final subgroup analysis.

We compared the choroidal vascular parameters between healthy participants and the two subgroups of individuals with schizophrenia, considering the duration of illness (DITYL and DIMTY) (Tables 4 and 5). Our results showed a significantly higher LA in the DIMTY group than in the control group (*p* = 0.001). There was no statistically significant difference in TCA, CVI, and SFCT between the DITYL, DIMTY, and healthy participants.

**Table 4:** The choroidal vascular profile in the DITYL, DIMTY, and healthy subjects.

	Group	Mean (SD)
LA (mm^2^)		
	Control	0.25 (0.04)
	DITYL	0.29 (0.02)
	DIMTY	0.30 (0.03)
TCA (mm^2^)		
	Control	0.41 (0.07)
	DITYL	0.44 (0.03)
	DIMTY	0.47 (0.06)
CVI		
	Control	0.60 (0.08)
	DITYL	0.66 (0.03)
	DIMTY	0.64 (0.01)
SFCT (µm)		
	Control	305.18 (43.13)
	DITYL	314.66 (13.50)
	DIMTY	309.23 (19.91)

**Table 5:** Comparison of choroidal vascular profile between the DITYL, DIMTY, and healthy subjects.

			Confidence interval		*p*
LA					
	Control	DITYL	−0.090	0.004	0.084
		DIMTY	−0.90	−0.022	0.001†
	DITYL	DIMTY	−0.037	0.060	0.802
TCA					
	Control	DITYL	−0.100	0.049	0.685
		DIMTY	−0.119	−0.005	0.029
	DITYL	DIMTY	−0.043	0.011	0.510
CVI					
	Control	DITYL	−0.131	0.022	0.207
		DIMTY	−0.090	0.026	0.384
	DITYL	DIMTY	−0.104	0.059	0.784
SFCT					
	Control	DITYL	−48.02	29.05	0.821
		DIMTY	−33.32	25.22	0.939
	DITYL	DIMTY	−46.73	35.86	0.945

^†^ Considered statistically significant.

Pearson's correlation analysis was utilized to investigate whether there is a correlation between the choroidal vascular parameters and the PANSS score. Our assessments showed no significant correlation (Table 6).

**Table 6:** Correlation analysis considering PANSS parameters score and choroidal vasculature.

		Luminal area (LA)	Total choroidal area (TCA)	Choroidal vascularity index (CVI)	Subfoveal choroidal thickness (SFCT)
PANSS positive scale	Pearson's correlation	−0.07	00	−0.22	−0.13
	*p* (two‐tailed)	0.73	1.00	0.33	0.57
PANSS negative scale	Pearson's correlation	−0.27	−0.21	−0.14	0.14
	*p* (two‐tailed)	0.23	0.35	0.55	0.52
Combination (positive–negative)	Pearson's correlation	0.18	0.19	−0.05	−0.23
	*p* (two‐tailed)	0.43	0.40	0.82	0.31
PANSS general psychopathology	Pearson's correlation	−0.25	−0.18	−0.16	−0.14
	*p* (two‐tailed)	0.23	0.43	0.48	0.54
PANSS anergia	Pearson's correlation	−0.29	−0.31	0.07	0.15
	*p* (two‐tailed)	0.19	0.16	0.87	0.52
PANSS thought disorder	Pearson's correlation	−0.20	−0.13	−0.17	−0.18
	*p* (two‐tailed)	0.372	0.55	0.46	0.43
PANSS activation	Pearson's correlation	0.26	0.29	−0.12	−0.4
	*p* (two‐tailed)	0.25	0.18	0.59	0.87
PANSS paranoid/belligerence	Pearson's correlation	−0.15	−0.03	−0.32	−0.04
	*p* (two‐tailed)	0.51	0.87	0.14	0.86
PANSS depression	Pearson's correlation	0.05	00	0.17	0.00
	*p* (two‐tailed)	0.80	1.00	0.45	1.00
PANSS total score	Pearson's correlation	−0.24	−0.16	−0.20	−0.04
	*p* (two‐tailed)	0.28	0.47	0.374	0.84

## 4 Discussion

In this study, we investigated the choroidal vasculature in patients with schizophrenia compared to healthy age and sex‐matched individuals. We showed that despite no statistically significant difference in the CT between the two groups of schizophrenia and healthy subjects, LA, TCA, and CVI were significantly higher in the schizophrenia group.

Increased CVI supports the hypothesis of microvascular dysfunction in schizophrenia. The precise mechanism and pathogenesis of microvascular changes in schizophrenia are unclear. Various hypotheses can be proposed to explain microvascular alterations in schizophrenia. Neurotransmitter imbalance, especially dopamine and glutamate, could alter the choroidal microcirculation homeostasis [28, 29]. Previous studies showed that dopamine modulates the pulsatile component of choroidal blood flow and is a crucial factor in ocular circulation [30]. Schizophrenia is widely regarded as a neurodevelopmental disorder, with disruptions in brain maturation contributing to its pathophysiology. Given the shared embryological origin and overlapping molecular signaling pathways between the brain and the eye—particularly the retina and choroid—ocular structures may also be affected in parallel. These similarities support the plausibility of detecting peripheral (ocular) biomarkers that reflect central (brain) pathology. However, the temporal and mechanistic relationship between neuronal dysfunction and choroidal vascular alterations remains uncertain [31, 32]. One proposed hypothesis regarding schizophrenia pathogenesis is the genetic‐inflammatory hypothesis [7, 33, 34]. An increase of immune/inflammatory genes linked with peripheral inflammatory indicators was found by ventricular choroidal plexus transcriptome analysis in schizophrenia. It was discovered that psychosis is associated with an increase in the choroid plexus volume with a favorable correlation with pro‐inflammatory cytokines [24]. Furthermore, anatomical and functional similarities exist between the eye choroid and the choroid plexus of the brain's ventricles. Different effects of inflammatory eye diseases on CVI have been reported [18, 35, 36]. This relation depends on the change ratio of the stromal part of the choroid to the luminal part of the vessels. The results of a study on obsessive–compulsive disorder patients showed an increase in CVI with a significant correlation with the inflammatory marker neutrophil‐to‐lymphocyte ratio in these patients [37]. Increasing the CVI in our study suggests that the effects are more prominent in LA in individuals with schizophrenia. In addition to these justifications, the role of metabolic profiles such as BMI, medications, and smoking in changes in choroidal blood supply should be mentioned [38, 39].

Findings from other psychiatric conditions support the relevance of inflammatory and oxidative mechanisms in our results. For example, studies demonstrating oxidative DNA damage and disturbed thiol/disulfide homeostasis in obsessive–compulsive disorder highlight systemic vulnerability to redox imbalance, a mechanism that is also implicated in schizophrenia. Similarly, investigations into kynurenine pathway metabolites in psychiatric disorders emphasize the contribution of immune–metabolic dysregulation, aligning with inflammatory hypotheses in schizophrenia [40, 41, 42, 43].

Evidence from retinal imaging studies in bipolar disorder further supports the value of ocular biomarkers in psychiatric research. The demonstration of retinal layer thinning in patients with bipolar disorder and their unaffected relatives suggests that structural ocular changes may reflect underlying neurobiological vulnerability—consistent with our findings of altered choroidal vascularity in schizophrenia [44, 45].

Additionally, psychodermatological studies on visible skin disorders, such as vitiligo and acne, illustrate how chronic psychological stress can modulate inflammatory pathways and oxidative processes. These insights are relevant because chronic stress, inflammation, and oxidative injury are interlinked components of schizophrenia pathophysiology that may contribute to microvascular dysfunction, including changes detectable in the choroid [46, 47, 48].

While our sample size constrained our division of patients based on the duration of illness, we were motivated by accumulating evidence that schizophrenia is a progressive neurovascular and neuroinflammatory disorder. Stratifying by illness duration allowed us to assess whether choroidal alterations reflect cumulative disease burden preliminarily. Future studies with larger sample sizes must validate this approach and better define duration‐related retinal biomarkers. Our results showed a significantly higher LA in both subgroups of individuals with schizophrenia regarding the disease chronicity (DIMTY and DITYL) than in healthy subjects. There was no statistically significant difference between the DITYL, DIMTY, and healthy participants in TCA, CVI, and SFCT.

Previous studies have reported several retinal abnormalities in individuals with schizophrenia. These include thinning of the retinal nerve fiber layer, decreased macular thickness, and altered retinal vascular caliber [13, 49, 50]. Electroretinographic studies have also shown reduced amplitude and delayed latency of both rod‐ and cone‐mediated responses, indicating possible retinal dopaminergic dysfunction [51]. These findings further support the hypothesis that structural and functional retinal alterations may mirror neurodevelopmental and neurodegenerative processes in the brain.

The assessment of choroidal alterations in schizophrenia has been the subject of a limited number of studies with inconsistent results. Li and colleagues found no significant differences in choroid vascular density and CT between patients with bipolar disorder, schizophrenia, and healthy controls [16]. However, the results of another study showed that patients with schizophrenia were shown to have higher choriocapillaris vascular density, particularly those in the early stages of the disease [52]. Demirlek and colleagues, in a study in 2023, showed that despite no significant difference in the CT in patients with the first episode of psychosis compared to healthy controls, CVI is significantly higher in these patients [25].

Most of the past studies on choroidal changes in schizophrenia were based on CT. CVI evaluation is more reliable than CT and is less affected by temporary local changes in blood flow. It also provides us with more data on the luminal and stromal components of the choroid [27, 53, 54].

The current study has some limitations. The cross‐sectional design and relatively small sample size lead to restrictions in the precise conclusion about the role of CVI as a biomarker of disease activity in schizophrenia. Conducting more studies with cohort design and larger sample sizes could help better understand the value of CVI as a biomarker in schizophrenia. Also, we did not include data on axial length, which could have impacted our results.

## 5 Conclusion

The results of this study showed a higher CVI value in patients with schizophrenia compared to healthy subjects. Increased CVI supports the hypothesis of microvascular dysfunction in schizophrenia. However, the precise mechanism and pathogenesis of microvascular changes in schizophrenia are unclear. We proposed neurotransmitter imbalance, especially dopamine and glutamate, and neuroinflammation as possible mechanisms of choroidal microcirculation abnormality. Our findings, considered alongside evidence from psychiatric, immunologic, and psychodermatological research, suggest that inflammation, oxidative stress, and chronic stress‐related biological changes may collectively contribute to the vascular alterations observed in schizophrenia, supporting the potential role of ocular imaging as a noninvasive biomarker in neuropsychiatric disease.

## Author Contributions

**Mehrdad Motamed Shariati:** conceptualization, methodology, software, data curation, investigation, formal analysis, supervision, writing – original draft, writing – review and editing. **Maryam Naghib:** data curation, investigation, writing – original draft. **Marziyeh Fotouhi:** data curation, investigation. **Mohammad Reza Fayyazi Bordbar:** conceptualization, methodology, writing – review and editing. All authors have read and approved the final version of the manuscript. Dr. Motamed Shariati had full access to all of the data in this study and took complete responsibility for the integrity of the data and the accuracy of the data analysis.

## Funding

The authors have nothing to report.

## Ethics Statement

The authors are accountable for all aspects of the work in ensuring that questions related to the accuracy or integrity of any part of the work are appropriately investigated and resolved. This study is done according to the Declaration of Helsinki and it was approved by the Institutional Review Board and Ethics Committee of the Mashhad University of Medical Sciences (Approval Number: IR.MUMS.MEDICAL.REC.1399.696).

## Consent

Informed consent for participation and publication was acquired from the patients or their legal guardians.

## Conflicts of Interest

The authors declare no conflicts of interest.

## Transparency Statement

The lead author Mehrdad Motamed Shariati affirms that this manuscript is an honest, accurate, and transparent account of the study being reported; that no important aspects of the study have been omitted; and that any discrepancies from the study as planned (and, if relevant, registered) have been explained.

## Data Availability

The data that support the findings of this study are available from the corresponding author upon reasonable request.

## References

[1] T. R. Insel , “Rethinking Schizophrenia,” Nature 468, no. 7321 (2010): 187–193. 21068826 10.1038/nature09552

[2] O. B. Smeland , O. Frei , A. M. Dale , and O. A. Andreassen , “The Polygenic Architecture of Schizophrenia—Rethinking Pathogenesis and Nosology,” Nature Reviews Neurology 16, no. 7 (2020): 366–379. 32528109 10.1038/s41582-020-0364-0

[3] W. S. Stone , M. R. Phillips , L. H. Yang , L. S. Kegeles , E. S. Susser , and J. A. Lieberman , “Neurodegenerative Model of Schizophrenia: Growing Evidence to Support a Revisit,” Schizophrenia Research 243 (2022): 154–162. 35344853 10.1016/j.schres.2022.03.004 PMC9189010

[4] K. Merritt , P. Luque Laguna , A. Irfan , and A. S. David , “Longitudinal Structural MRI Findings in Individuals at Genetic and Clinical High Risk for Psychosis: A Systematic Review,” Frontiers in Psychiatry 12 (2021): 49. 10.3389/fpsyt.2021.620401 PMC7884337 33603688

[5] S. Cetin‐Karayumak , M. A. Di Biase , N. Chunga , et al., “White Matter Abnormalities Across the Lifespan of Schizophrenia: A Harmonized Multi‐Site Diffusion MRI Study,” Molecular Psychiatry 25, no. 12 (2020): 3208–3219. 31511636 10.1038/s41380-019-0509-y PMC7147982

[6] N. F. Ho , P. L. H. Chong , D. R. Lee , Q. H. Chew , G. Chen , and K. Sim , “The Amygdala in Schizophrenia and Bipolar Disorder: A Synthesis of Structural MRI, Diffusion Tensor Imaging, and Resting‐State Functional Connectivity Findings,” Harvard Review of Psychiatry 27, no. 3 (2019): 150–164. 31082993 10.1097/HRP.0000000000000207

[7] D. R. Hanson and I. I. Gottesman , “Theories of Schizophrenia: A Genetic‐Inflammatory‐Vascular Synthesis,” BMC Medical Genetics 6, no. 1 (2005): 7. 15707482 10.1186/1471-2350-6-7 PMC554096

[8] G. Fond , C. Lançon , T. Korchia , P. Auquier , and L. Boyer , “The Role of Inflammation in the Treatment of Schizophrenia,” Frontiers in Psychiatry 11 (2020): 518291. 10.3389/fpsyt.2020.00160 PMC7093323 32256401

[9] R. Upthegrove and G. M. Khandaker , “Cytokines, Oxidative Stress and Cellular Markers of Inflammation in Schizophrenia,” in Neuroinflammation and Schizophrenia, Current Topics in Behavioral Neurosciences, eds. G. Khandaker , U. Meyer , P. Jones , 44 (Springer, 2019), 49–66, 10.1007/7854_2018_88Reference31. 31115797

[10] O. P. du Sert , J. Unrau , C. J. Gauthier , et al., “Cerebral Blood Flow in Schizophrenia: A Systematic Review and Meta‐Analysis of MRI‐Based Studies,” Progress in Neuro‐Psychopharmacology and Biological Psychiatry 121 (2023): 110669. 36341843 10.1016/j.pnpbp.2022.110669

[11] C. S. Legind , B. V. Broberg , R. Brouwer , et al., “Heritability of Cerebral Blood Flow and the Correlation to Schizophrenia Spectrum Disorders: A Pseudo‐Continuous Arterial Spin Labeling Twin Study,” Schizophrenia Bulletin 45, no. 6 (2019): 1231–1241. 30776063 10.1093/schbul/sbz007 PMC6811820

[12] N. Sukumar , P. Sabesan , U. Anazodo , and L. Palaniyappan , “Neurovascular Uncoupling in Schizophrenia: A Bimodal Meta‐Analysis of Brain Perfusion and Glucose Metabolism,” Frontiers in Psychiatry 11 (2020): 754. 32848931 10.3389/fpsyt.2020.00754 PMC7427579

[13] R. Daneshvar , M. Naghib , M. R. Fayyazi Bordbar , F. Faridhosseini , M. Fotouhi , and M. Motamed Shariati , “Optic Nerve Head Neurovascular Assessments in Patients With Schizophrenia: A Cross‐Sectional Study,” Health Science Reports 7, no. 5 (2024): e2100. 38725558 10.1002/hsr2.2100 PMC11079145

[14] S. M. Silverstein , A. Lai , K. M. Green , C. Crosta , S. I. Fradkin , and R. S. Ramchandran , “Retinal Microvasculature in Schizophrenia,” Eye and Brain 13 (2021): 205–217. 34335068 10.2147/EB.S317186 PMC8318708

[15] K. A. Douglas , D. Bannai , I. Adhan , et al., “A Preliminary Study Using OCT‐A to Determine Deep Layer Retinal Vascular Changes in Schizophrenia,” Biological Psychiatry 87, no. 9 (2020): S244–S245.

[16] C. Y. Li , I. Garg , D. Bannai , et al., “Sex‐Specific Changes in Choroid Vasculature Among Patients With Schizophrenia and Bipolar Disorder,” Clinical Ophthalmology 16 (2022): 2363–2371. 35924185 10.2147/OPTH.S352731 PMC9343178

[17] C. Iovino , M. Pellegrini , F. Bernabei , et al., “Choroidal Vascularity Index: An In‐Depth Analysis of This Novel Optical Coherence Tomography Parameter,” Journal of Clinical Medicine 9, no. 2 (2020): 595. 32098215 10.3390/jcm9020595 PMC7074450

[18] R. Agrawal , J. Ding , P. Sen , et al., “Exploring Choroidal Angioarchitecture in Health and Disease Using Choroidal Vascularity Index,” Progress in Retinal and Eye Research 77 (2020): 100829. 31927136 10.1016/j.preteyeres.2020.100829

[19] A. B. Schroeder , E. T. A. Dobson , C. T. Rueden , P. Tomancak , F. Jug , and K. W. Eliceiri , “The ImageJ Ecosystem: Open‐Source Software for Image Visualization, Processing, and Analysis,” Protein Science 30, no. 1 (2021): 234–249. 33166005 10.1002/pro.3993 PMC7737784

[20] O. Senay , M. Seethaler , N. Makris , et al., “A Preliminary Choroid Plexus Volumetric Study in Individuals With Psychosis,” Human Brain Mapping 44, no. 6 (2023): 2465–2478. 36744628 10.1002/hbm.26224 PMC10028672

[21] P. Lizano , S. Pong , S. Santarriaga , D. Bannai , and R. Karmacharya , “Brain Microvascular Endothelial Cells and Blood‐Brain Barrier Dysfunction in Psychotic Disorders,” Molecular Psychiatry 28, no. 9 (2023): 3698–3708. 37730841 10.1038/s41380-023-02255-0

[22] B. K. Y. Bitanihirwe , P. Lizano , and T. U. W. Woo , “Deconstructing the Functional Neuroanatomy of the Choroid Plexus: An Ontogenetic Perspective for Studying Neurodevelopmental and Neuropsychiatric Disorders,” Molecular Psychiatry 27, no. 9 (2022): 3573–3582. 35618887 10.1038/s41380-022-01623-6 PMC9133821

[23] D. Bannai , M. Reuter , R. Hegde , et al., “Linking Enlarged Choroid Plexus With Plasma Analyte and Structural Phenotypes in Clinical High Risk for Psychosis: A Multisite Neuroimaging Study,” Brain, Behavior, and Immunity 117 (2024): 70–79. 38169244 10.1016/j.bbi.2023.12.021 PMC10932816

[24] P. Lizano , O. Lutz , G. Ling , et al., “Association of Choroid Plexus Enlargement With Cognitive, Inflammatory, and Structural Phenotypes Across the Psychosis Spectrum,” American Journal of Psychiatry 176, no. 7 (2019): 564–572. 31164007 10.1176/appi.ajp.2019.18070825 PMC6676480

[25] C. Demirlek , F. Atas , B. Yalincetin , et al., “Choroidal Structural Analysis in Ultra‐High Risk and First‐Episode Psychosis,” European Neuropsychopharmacology 70 (2023): 72–80. 36931136 10.1016/j.euroneuro.2023.02.016

[26] M. M. Shariati and N. Shoeibi , “Image Calibration in Choroidal Vascularity Index Measurement,” International Journal of Retina and Vitreous 9, no. 1 (2023): 66. 37950306 10.1186/s40942-023-00503-7 PMC10636868

[27] R. Agrawal , P. Gupta , K.‐A. Tan , C. M. G. Cheung , T.‐Y. Wong , and C.‐Y. Cheng , “Choroidal Vascularity Index as a Measure of Vascular Status of the Choroid: Measurements in Healthy Eyes From a Population‐Based Study,” Scientific Reports 6, no. 1 (2016): 21090. 26868048 10.1038/srep21090 PMC4751574

[28] K.‐H. Huemer , C. Zawinka , G. Garhofer , et al., “Effects of Dopamine on Retinal and Choroidal Blood Flow Parameters in Humans,” British Journal of Ophthalmology 91, no. 9 (2007): 1194–1198. 17383995 10.1136/bjo.2006.113399 PMC1954943

[29] J. Harned , S. Nagar , and M. C. McGahan , “Hypoxia Controls Iron Metabolism and Glutamate Secretion in Retinal Pigmented Epithelial Cells,” Biochimica et Biophysica Acta (BBA)‐General Subjects 1840, no. 10 (2014): 3138–3144. 24972165 10.1016/j.bbagen.2014.06.012

[30] H. A. Reitsamer , C. Zawinka , and M. Branka , “Dopaminergic Vasodilation in the Choroidal Circulation by d1/d5 Receptor Activation,” Investigative Ophthalmology & Visual Science 45, no. 3 (2004): 900–905. 14985308 10.1167/iovs.03-0997

[31] D. Weinberger and S. Marenco , “Schizophrenia as a Neurodevelopmental Disorder,” in Schizophrenia (2003): 326–348.

[32] J. Graw , “Eye Development,” Current Topics in Developmental Biology 90 (2010): 343–386. 20691855 10.1016/S0070-2153(10)90010-0

[33] N. Müller , E. Weidinger , B. Leitner , and M. J. Schwarz , “The Role of Inflammation in Schizophrenia,” Frontiers in Neuroscience 9 (2015): 372. 26539073 10.3389/fnins.2015.00372 PMC4612505

[34] B. J. Miller and D. R. Goldsmith , “Evaluating the Hypothesis That Schizophrenia Is an Inflammatory Disorder,” Focus 18, no. 4 (2020): 391–401. 33343251 10.1176/appi.focus.20200015 PMC7725151

[35] A. Baytaroğlu , S. Kadayifçilar , A. Ağin , et al., “Choroidal Vascularity Index as a Biomarker of Systemic Inflammation in Childhood Polyarteritis Nodosa and Adenosine Deaminase‐2 Deficiency,” Pediatric Rheumatology 18, no. 1 (2020): 1–10. 32245490 10.1186/s12969-020-0417-3 PMC7118843

[36] M. Kim , R. Y. Kim , and Y.‐H. Park , “Choroidal Vascularity Index and Choroidal Thickness in Human Leukocyte Antigen‐B27‐Associated Uveitis,” Ocular Immunology and Inflammation 27, no. 8 (2019): 1280–1287, 10.1080/09273948.2018.1530364. 30285514

[37] B. Sekeryapan Gediz , M. Ozturk , H. Kilinc Hekimsoy , E. G. Yuksel , and Y. Ozdamar Erol , “Choroidal Vascularity Index as a Potential Inflammatory Biomarker for Obsessive Compulsive Disorder,” Ocular Immunology and Inflammation 30, no. 2 (2022): 428–432. 32946294 10.1080/09273948.2020.1800052

[38] X. Wei , S. Kumar , J. Ding , N. Khandelwal , M. Agarwal , and R. Agrawal , “Choroidal Structural Changes in Smokers Measured Using Choroidal Vascularity Index,” Investigative Ophthalmology & Visual Science 60, no. 5 (2019): 1316–1320. 30943279 10.1167/iovs.18-25764

[39] N. Aşıkgarip , E. Temel , A. Kıvrak , and K. Örnek , “Choroidal Structural Changes and Choroidal Vascularity Index in Patients With Systemic Hypertension,” European Journal of Ophthalmology 32, no. 4 (2022): 2427–2432. 34313148 10.1177/11206721211035615

[40] D. Sonmez , F. Kurhan , and C. Hocaoglu , “Neurobiology of Suicide in Depressive Disorders,” in Handbook of the Biology and Pathology of Mental Disorders (Springer International Publishing, 2024), 1–24.

[41] F. Kurhan , G. Z. Kamis , H. H. Alp , E. F. Akyuz Cim , and A. Atli , “A Cross‐Sectional Measurement of Endogenous Oxidative Stress Marker Levels in Obsessive Compulsive Disorder,” Psychiatry and Clinical Psychopharmacology 32, no. 3 (2022): 215–221. 38766666 10.5152/pcp.2022.21318 PMC11099616

[42] F. Kurhan , H. H. Alp , M. Işık , and Y. S. Atan , “The Evaluation of Thiol/Disulfide Homeostasis and Oxidative DNA Damage in Patients With Obsessive Compulsive Disorder,” Clinical Psychopharmacology and Neuroscience 20, no. 2 (2022): 240–247. 35466095 10.9758/cpn.2022.20.2.240 PMC9048000

[43] H. H. Alp , F. Kurhan , and H. İ. Akbay , “Predictive Value of Kynurenine Pathway Metabolites in the Severity of Patients With Obsessive‐Compulsive Disorder,” Psychiatry and Clinical Neurosciences 79, no. 7 (2025): 378–388. 40167157 10.1111/pcn.13819

[44] F. Kurhan , V. Yıldız , G. Z. Kamış , K. Karataş , and M. Batur , “Evaluation of the Electroconvulsive Therapy's Impact on Retinal Structures in First‐Episode Psychosis Patients Using Optical Coherence Tomography,” Schizophrenia Bulletin 52, no. 2 (2024): sbae187. 10.1093/schbul/sbae187 PMC12996877 39591543

[45] E. Seven and F. Kurhan , “Evaluation of Retinal Layer Thickness in Patients With Bipolar Disorder, Their Relatives, and Healthy Controls Using Optical Coherence Tomography,” World Journal of Biological Psychiatry 26, no. 6 (2025): 224–233. 10.1080/15622975.2025.2505148 40401999

[46] F. Kurhan and İ. H. Yavuz , “The Impact of Facial Vitiligo on Social Appearance Anxiety: A Case‐Control Study,” Clinical and Experimental Dermatology 51, no. 3 (2025): llaf471. 10.1093/ced/llaf471 41134691

[47] A. Yılgör , F. Kurhan , and A. Yalın , “Sleep, Rhythm, and Mental Burden: Disruption of Psychobiological Balance in Patients With Myasthenia Gravis,” Biological Rhythm Research 57, no. 4 (2025): 1–5.

[48] F. Kurhan , M. Arslan , and Ç. Hocaoğlu , “Obsessive‐Compulsive Disorder and DNA Damage,” in Handbook of the Biology and Pathology of Mental Disorders (Springer International Publishing, 2024), 1–26.

[49] M. M. Shariati and A. Darvish , “Optic Nerve Head and Macular Neurovasculature in Psychosis,” Journal of Clinical Images and Medical Case Reports 4, no. 6 (2023): 2475.

[50] H. Komatsu , G. Onoguchi , S. M. Silverstein , et al., “Retina as a Potential Biomarker in Schizophrenia Spectrum Disorders: A Systematic Review and Meta‐Analysis of Optical Coherence Tomography and Electroretinography,” Molecular Psychiatry 29, no. 2 (2024): 464–482. 38081943 10.1038/s41380-023-02340-4 PMC11116118

[51] D. L. Demmin , Q. Davis , M. Roché , and S. M. Silverstein , “Electroretinographic Anomalies in Schizophrenia,” Journal of Abnormal Psychology 127, no. 4 (2018): 417–428. 29745706 10.1037/abn0000347

[52] D. Bannai , I. Adhan , R. Katz , et al., “Quantifying Retinal Microvascular Morphology in Schizophrenia Using Swept‐Source Optical Coherence Tomography Angiography,” Schizophrenia Bulletin 48, no. 1 (2021): 80–89. 10.1093/schbul/sbab111 PMC8781445 34554256

[53] K. Breher , L. Terry , T. Bower , and S. Wahl , “Choroidal Biomarkers: A Repeatability and Topographical Comparison of Choroidal Thickness and Choroidal Vascularity Index in Healthy Eyes,” Translational Vision Science & Technology 9, no. 11 (2020): 8. 10.1167/tvst.9.11.8 PMC7552934 33133771

[54] M. Motamed Shariati , S. Khazaei , and M. Yaghoobi , “Choroidal Vascularity Index in Health and Systemic Diseases: A Systematic Review,” International Journal of Retina and Vitreous 10, no. 1 (2024): 87. 39558436 10.1186/s40942-024-00607-8 PMC11575059

